# Functional Analysis of Casein Kinase 1 in a Minimal Circadian System

**DOI:** 10.1371/journal.pone.0070021

**Published:** 2013-07-25

**Authors:** Gerben van Ooijen, Matthew Hindle, Sarah F. Martin, Martin Barrios-Llerena, Frédéric Sanchez, François-Yves Bouget, John S. O’Neill, Thierry Le Bihan, Andrew J. Millar

**Affiliations:** 1 SynthSys, University of Edinburgh, Edinburgh, United Kingdom; 2 Institute for Molecular Plant Sciences, University of Edinburgh, Edinburgh, United Kingdom; 3 Institute of Structural and Molecular Biology, University of Edinburgh, Edinburgh, United Kingdom; 4 Centre National de la Recherche Scientifique, Université Pierre et Marie Curie, Paris, France; 5 Laboratoire d’Océanographie Microbienne, Observatoire Océanologique, Banyuls-sur-Mer, France; 6 Medical Research Council Laboratory for Molecular Biology, Cambridge, United Kingdom; Simon Fraser University, Canada

## Abstract

The Earth’s rotation has driven the evolution of cellular circadian clocks to facilitate anticipation of the solar cycle. Some evidence for timekeeping mechanism conserved from early unicellular life through to modern organisms was recently identified, but the components of this oscillator are currently unknown. Although very few clock components appear to be shared across higher species, Casein Kinase 1 (CK1) is known to affect timekeeping across metazoans and fungi, but has not previously been implicated in the circadian clock in the plant kingdom. We now show that modulation of CK1 function lengthens circadian rhythms in *Ostreococcus* *tauri* , a unicellular marine algal species at the base of the green lineage, separated from humans by ~1.5 billion years of evolution. CK1 contributes to timekeeping in a phase-dependent manner, indicating clock-mediated gating of CK1 activity. Label-free proteomic analyses upon overexpression as well as inhibition revealed CK1-responsive phosphorylation events on a set of target proteins, including highly conserved potentially clock-relevant cellular regulator proteins. These results have major implications for our understanding of cellular timekeeping and can inform future studies in any circadian organism.

## Introduction

The circadian clock is a timekeeping mechanism that evolved to adapt to, and anticipate the predictable daily changes associated with the Earth’s rotation. In many organisms, genes have been identified whose products are rhythmically expressed and subsequently drive rhythms in the activity of other genes, that can directly or indirectly feed back to form loops that oscillate in a ~24 hour rhythm [1,2]. These so-called transcriptional-translational feedback loops, or TTFLs, are identified in most model organisms whose clock has been studied in any detail, and were long believed to be the sole drivers of cellular circadian rhythmicity.

Indeed, deletion of TTFL function leads to elimination of the majority of clock outputs, both transcriptional and physiological. However, in cyanobacteria, a circadian oscillator was identified that did not rely upon rhythmic transcription, and is based upon cycles of phosphorylation and de-phosphorylation of the protein kinase KaiC, regulated by two other proteins, KaiA and KaiB [3-5]. Strikingly, these three protein continue to oscillate outside their cellular context, since cycles of KaiC phosphorylation can be reconstituted in vitro using only the recombinant Kai proteins with ATP [6].

These Kai proteins are not conserved in eukaryotes, leading many to believe that non-transcriptional oscillators (NTOs) were an exclusively prokaryotic phenomenon. Additionally, actual TTFL components are not conserved across higher taxa either, although the overall topology of the TTFL circuitry is remarkably similar between species [2]. This lack of conservation led to the assumption that timekeeping evolved multiple times, convergently.

Recently however, evidence for the presence of a timekeeping mechanism conserved across an unprecedentedly diverse array of species was observed. A rhythmic clock-regulated output in the oxidation state of 2-Cys peroxiredoxin (PRX) proteins was identified that did not depend on active transcriptional timekeeping [7,8]. PRX rhythms were subsequently shown to be shared from Archaea, to fungi, to plants, to animals [9,10]. These findings lend a fresh perspective to research into clock mechanisms and pose a key question concerning the identity of the cellular components which contribute to this novel timekeeping mechanism [11].

Myriad studies have shown TTFL protein activity to be regulated post-translationally [12-14], and Casein Kinase 1 (CK1) is one of the few proteins known to affect timekeeping across species [11]. Other than circadian rhythms, CK1 has a wide range of cellular functions, regulating processes as diverse as membrane trafficking, DNA replication, wnt signalling, and RNA metabolism [15]. CK1 isoforms affect rhythmicity in animal species [16-18] as well as in the fungus *Neurospora crassa* [19], although the known target proteins of CK1 within the TTFL systems are not conserved between animals and fungi. In animals, the most prominent clock-relevant CK1 target is the Period (PER) protein, whose rhythmic phosphorylation by CK1 regulates PER stability and sub-cellular localisation [18,20-23]. In *Neurospora* , rhythmic interaction of CK1 with Frequency (FRQ) leads to hyper-phosphorylation of FRQ, resulting in degradation [19]. For both PER and FRQ genomic loci, the cognate transcriptional activators are also directly regulated by CK1 (i.e. the Clock/BMAL complex in animals [24] and the White Collar Complex (WCC) in fungi [25]). In spite of the remarkable conservation of CK1 isoforms in the green lineage of evolution, no contribution to timekeeping by any CK1 orthologs have been reported to date.

The marine unicellular algal species *Ostreococcus* *tauri* is a primitive green organism similar to the ancestor of modern land plants, and separated from the metazoan lineage, including humans, by an estimated 1.5 billion years [26]. This novel model organism of greatly reduced genomic [27] and cellular complexity [28] is ideally suited to the analysis of cellular systems which have proven difficult to address in more complex eukaryotic model organisms such as the mouse, fruit-fly, *Neurospora* , or *Arabidopsis* .

The aim of this study was to employ *O* *. tauri* as a minimal circadian model organism to identify potential functional conservation of CK1 in timekeeping in the plant kingdom. Significantly, this simple picoeukaryote has been already been employed to probe circadian clock dynamics with great success, since in spite of its tiny genome, *O* *. tauri* contains fully functional TTFL and non-transcriptional circadian systems [8,29-32].

Using genetic overexpression and pharmacological inhibition we show that CK1 indeed contributes to timekeeping in the green lineage. Furthermore, phospho-proteomic analyses performed at the CK1 peak phase resulted in a list of potential clock-relevant CK1 targets, most of which are conserved across taxa and some of which have confirmed circadian roles in other organisms.

These results introduce CK1 as one of the most ancient clock components known to date, and imply that ancient post-translational regulatory proteins may well constitute a basic and ancient level of timekeeping in modern organisms.

## Results

### CK1 represented by a single protein in *O* *. tauri*

**Figure 1:**
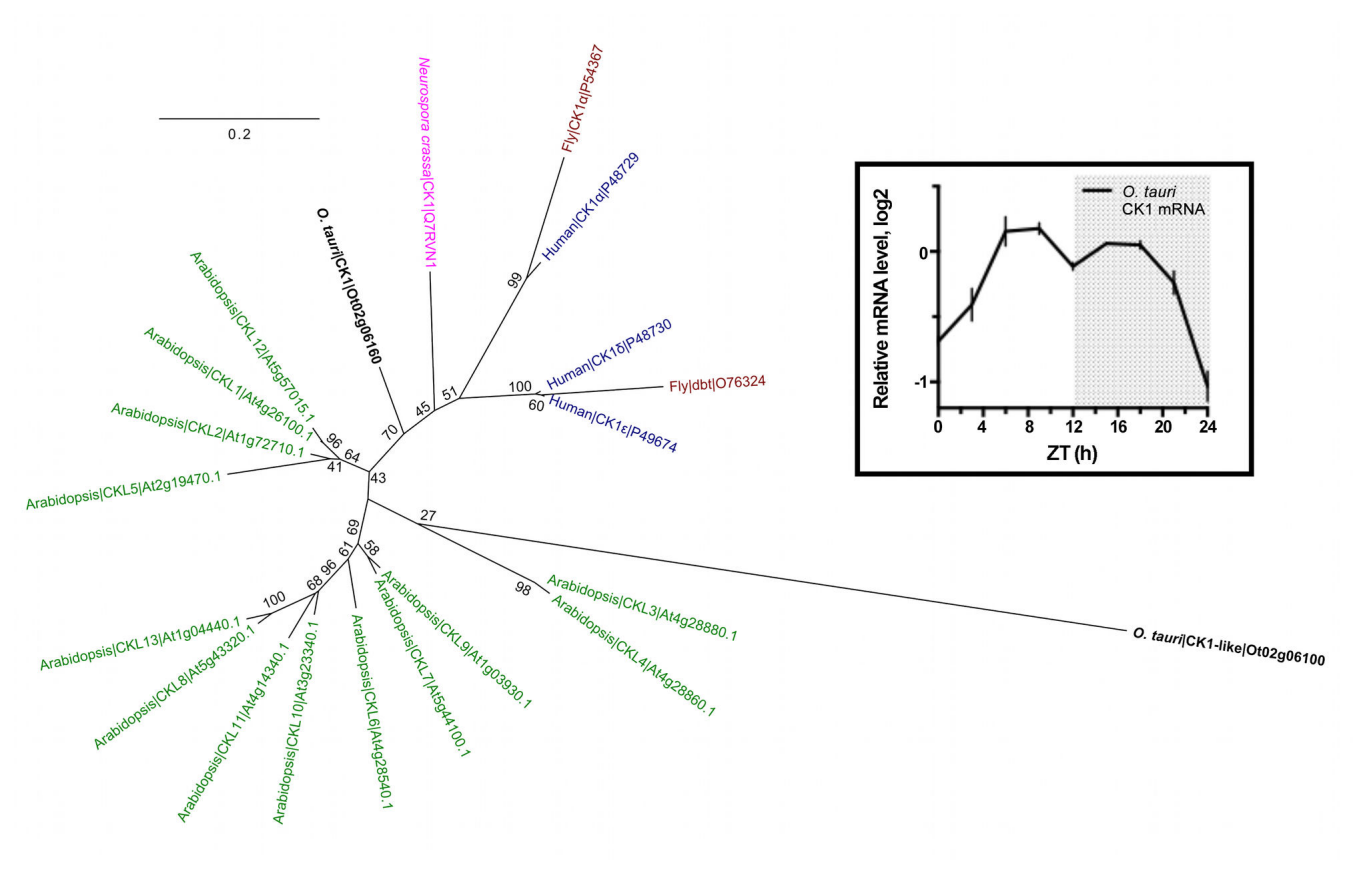
The single CK1 homolog in *O* *. tauri* is diurnally expressed. A rooted phylogenetic tree has been generated from a sequence alignment of the CK1 isoforms in animal species *Drosophila melanogaster*, human (or mouse), the fungus *Neurospora crassa*, the moss *Physcomitrella* *patens* , the plant *Arabidopsis thaliana*, and the green alga *Ostreococcus* *tauri* . A single CK1 homolog was identified in *O* *. tauri* , that was identified from publicly available microarray data as diurnally regulated (insert, top right). The next-closest CK1-like protein from *O* *. tauri* (Ot02g06100) was used as the out-group.

Most organisms contain several isoforms of CK1, each with different substrate specificities. Animal isoforms epsilon, alpha and delta all contribute to timekeeping [16,17]. Mouse isoforms are identical to human isoforms in the aligned sequences underlying this tree. In the unicellular eukaryotic model organism *Ostreococcus* *tauri* , a single CK1 protein exists (Figure 1) with remarkable homology to human clock-relevant isoforms CK1δ (72% maximum identity), CK1ε (71%) and CK1α (70%). From publicly available micro-array data [30] it is evident that in *O* *. tauri* , this single CK1 homolog is diurnally regulated with peak expression around the light/dark transition at dusk (Figure 1). To verify that only one CK1 exists, the next closest sequence in *O* *. tauri* was included and this protein forms a distant outgroup branching off from the plant expansion. In the model plant *Arabidopsis thaliana*, CK1 diversified into at least 12 members, none of which have identified clock function. None of CK1’s identified animal or fungal TTFL protein targets PER, FRQ, Clock, and WC2 are conserved in *O* *. tauri* , nor in higher plants evolved from this branch.

### Long-period rhythms upon CK1 overexpression

**Figure 2:**
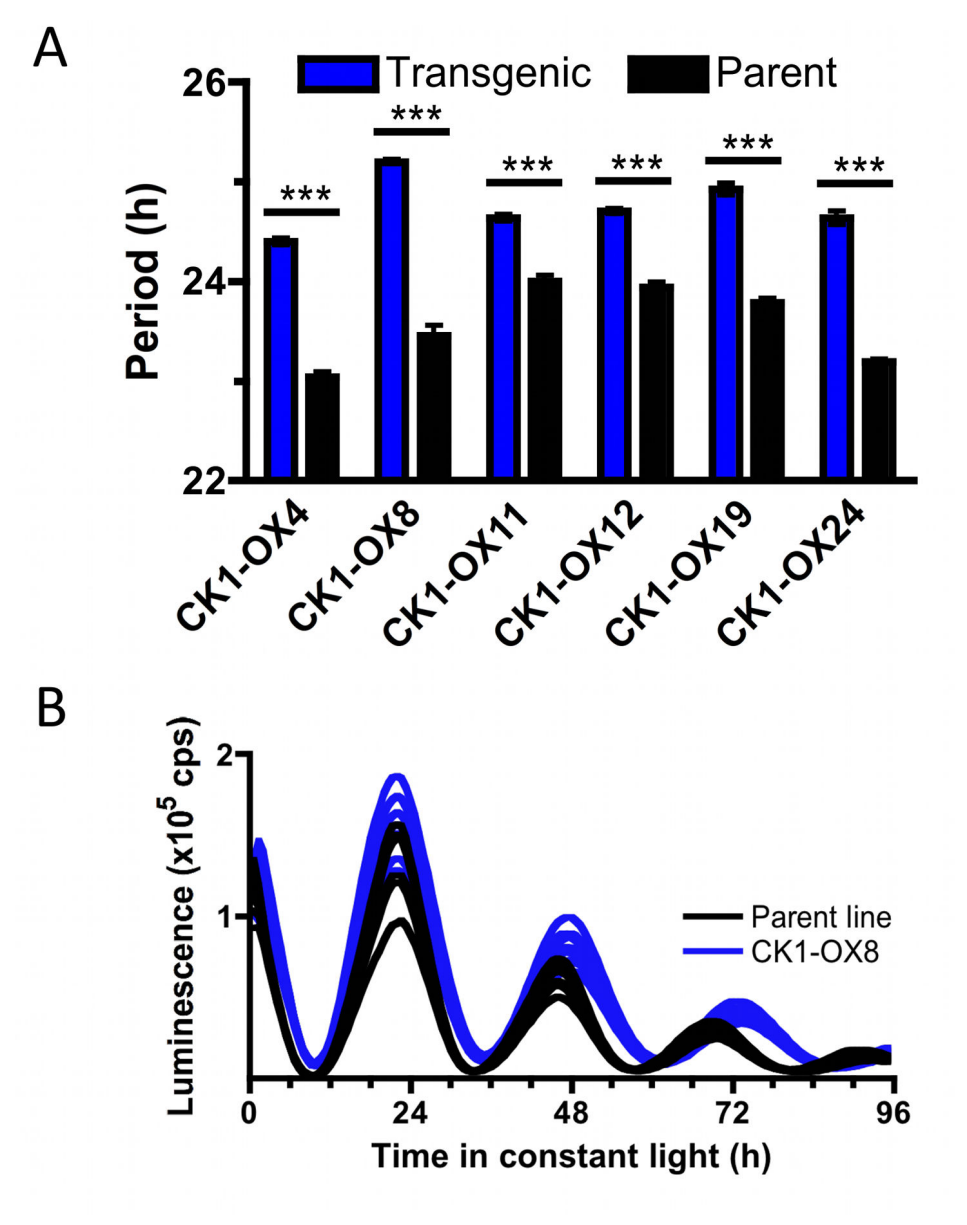
Long period phenotype induced by CK1 overexpression. (**A**) Free-running period was analysed by bioluminescent imaging in 6 independent transgenic lines overexpressing CK1 in the CCA1-LUC background. Lines were compared against the parent line in identical plate positions. In all cases, a significantly (p<0.001) long circadian period was observed (n=8). (**B**) Example traces of luminescent lines overexpressing CK1 (CK1-OX8, blue) compared to the parent line CCA1-LUC (black) in constant light.

To test conserved clock function of CK1, we transformed *O* *. tauri* cells [33] carrying a rhythmically luminescent reporter (CCA1-LUC) [29] with an overexpression construct of CK1 containing a selectable marker. The effect of overexpression on circadian period was analysed by bioluminescent imaging in constant light. A long-period phenotype was associated with CK1 overexpression in all six verified independent transgenic lines (Figure 2A) as visualised by the CCA1-LUC construct (Figure 2B), strongly indicating CK1 functions in timekeeping in this organism in spite of the absence of known clock-relevant CK1 targets identified in other taxa.

### Inhibition of CK1 activity influences *O* *. tauri* timekeeping

**Figure 3:**
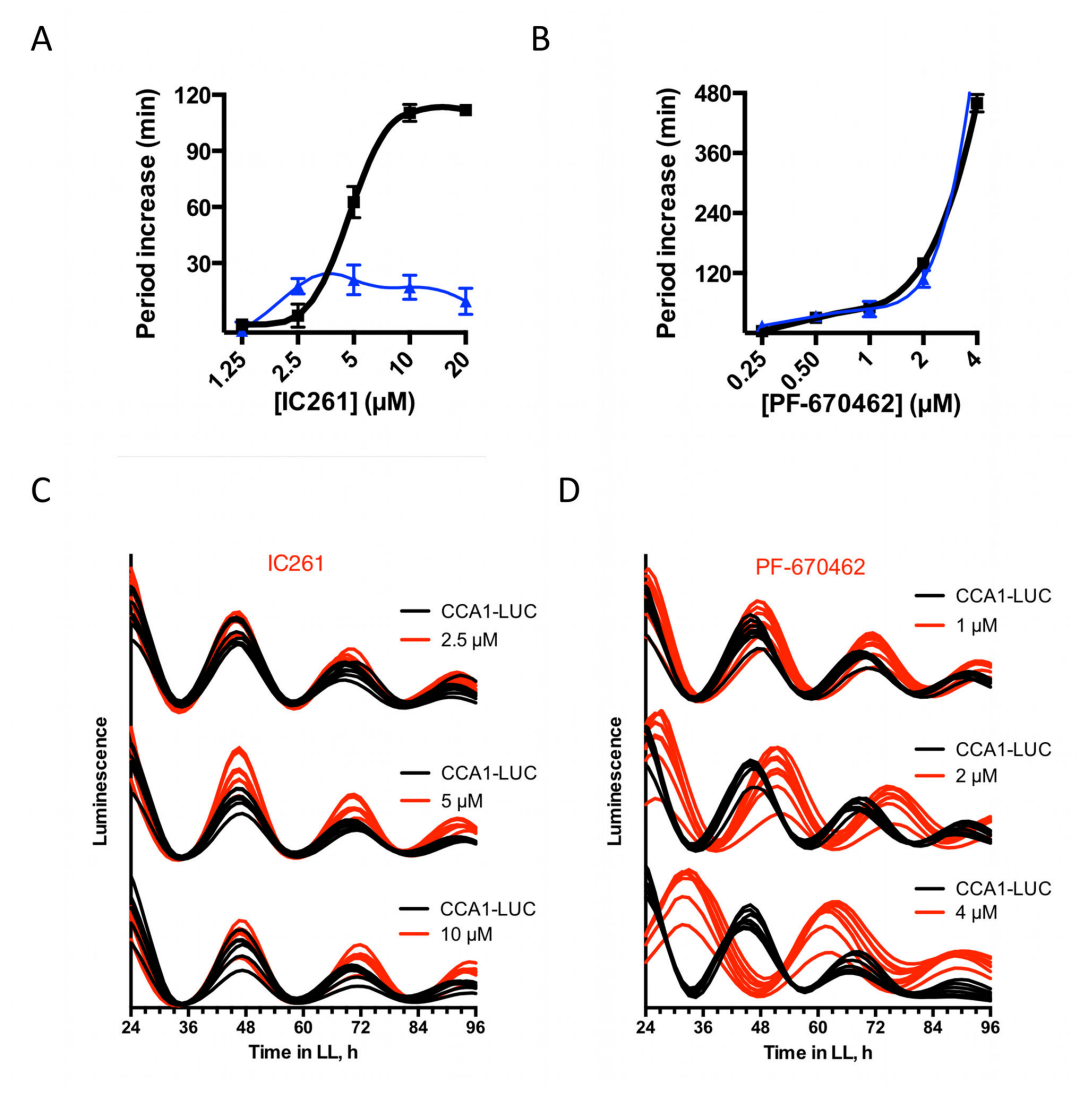
CK1 inhibitor leads to period lengthening. (**A**–**B**) Dose–response curves showing free-running period lengthening of CCA1-LUC lines (black line) treated with CK1 inhibitor IC261 (A) or PF-670462 (B). Blue line indicates the period effects of the same range of inhibitors on the CK1 overexpression line CK1-OX8. (**C**–**D**) For relevant drug concentrations, the raw data is provided for indicated inhibitor, compared to vehicle-treated CCA1-LUC parent cells (red traces are treated, black traces mock-treated, n=8).

To further substantiate a functionally conserved role of CK1 in circadian timekeeping in *O* *. tauri* , chemical inhibition of CK1 was employed. Inhibition using IC261 or PF-670462 was reported to lengthen circadian period in various model species [8,34-36]. Indeed, CK1 inhibition with either drug similarly induced dose-dependent period lengthening in CCA1-LUC cells: Treatment with IC261 dose-dependently increased circadian period with two hours (Figure 3A, C). When the overexpression line CK1-OX8 was subjected to the same concentration range of inhibitor, this period-lengthening effect was lost (Figure 3A), providing evidence that IC261 indeed hits *O* *. tauri* CK1 (the only target protein that has meaningful homology to human CK1 present in the genome).

CK1 inhibitor PF-670462 increased circadian period in a dose-dependent manner by a maximum of 7.7 hours (±0.3, n=8, Figure 3B, D), and the CK1-OX8 line is similarly susceptible to this inhibitor. The higher period-lengthening effect and similar susceptibility of the overexpression line could either indicate that PF-670462 also acts on secondary targets that affect the *O* *. tauri* clock, or that IC-261 only leads to partial inhibition of CK1 and that inhibitory action of PF-670462 is too strong to overcome by overexpression.

### Differential phase-shifting by CK1 inhibition

**Figure 4:**
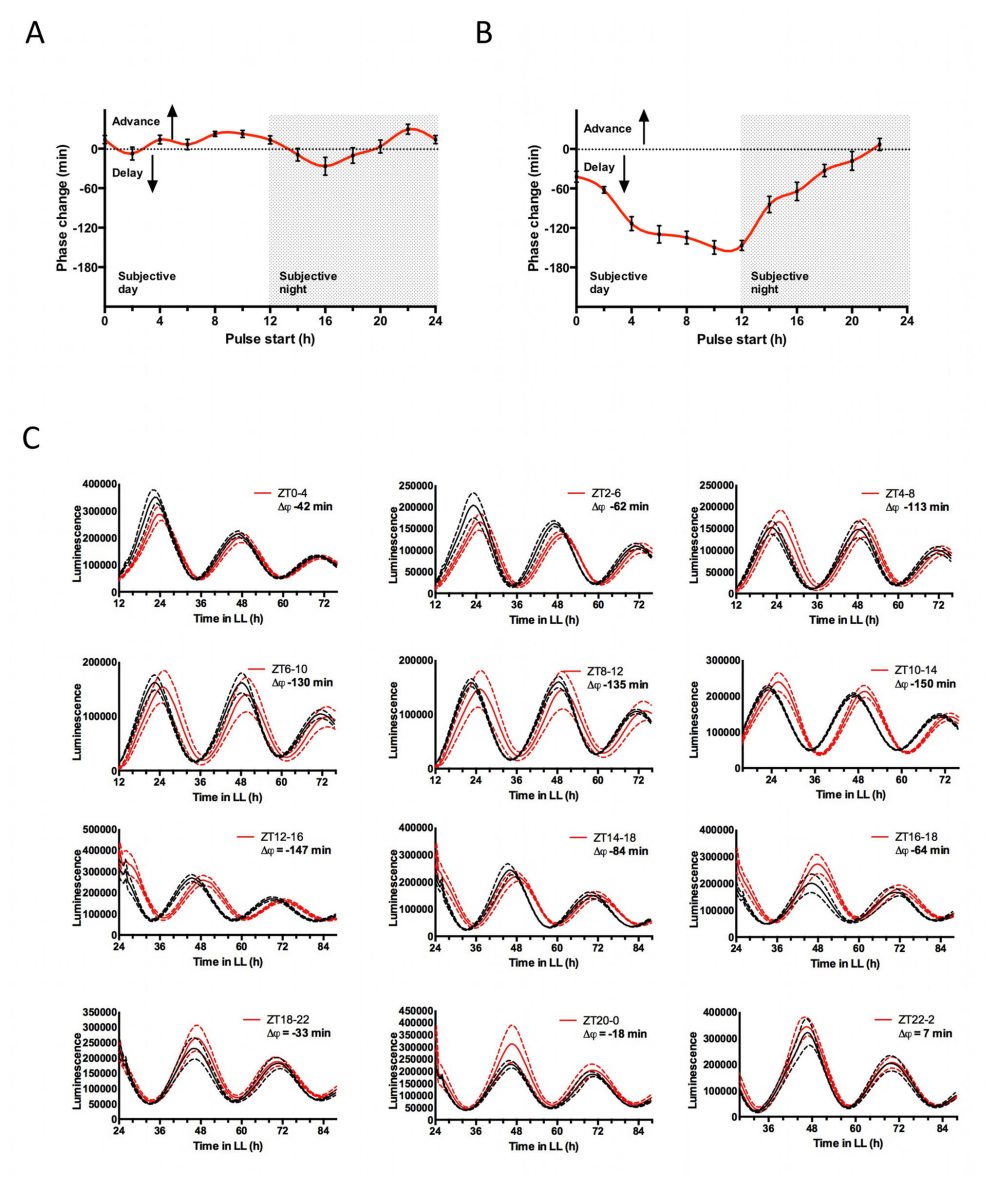
Phase-dependent effect of CK1 inhibition pulses. (**A**–**B**) Phase-response curves of CCA1-LUC cells to 4-hour treatment pulses with 10 µM CK1 inhibitor IC261 (A) or 4 µM PF-670462 (B). The start time of the pulse (hours after transfer to constant light) is plotted against the phase change of reinitiated circadian rhythms observed upon wash-off of the inhibitor, compared to vehicle-treated cells (n=8). (**C**) Traces of rhythms appearing after vehicle treatments (black traces) versus PF-670462-treated cells (red), for all treatments. Dotted lines indicate SD (n=8). Treatment times (in ZT) are provided, along with approximate average phase change (Δϕ) in minutes. Phase delays are negative values by consensus.

**Figure 5:**
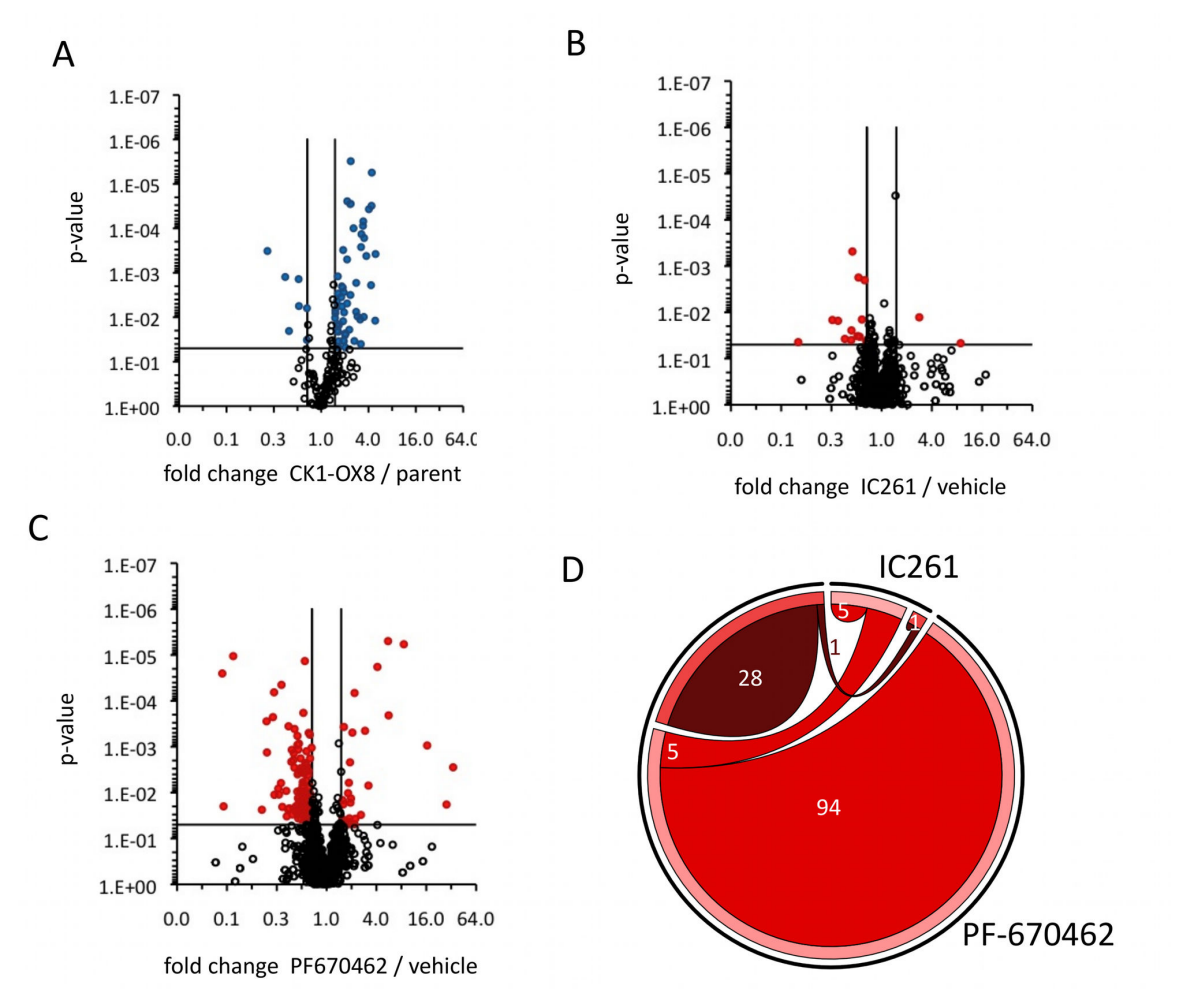
Changes to the phospho-proteome upon CK1 modulation. (**A**) Volcano plot visualising quantified phospho-sites with significant differences between CK1-OX8 and CCA1-LUC in a pair-wise comparison, using label-free quantification by mass spectrometry. Criteria were p<0.05 and fold changes >1.5 or <0.67, as indicated by black lines. Blue data-points represent significantly differential phospho-sites, black circles represent those that do not change significantly. The identities of these sites can be found in Supplementary Dataset S1. (**B**–**C**) Volcano plots visualising quantified phospho-sites with significant differences between mock-treated cells and cells inhibited for CK1 using IC-261 (B) or PF-670462 (C) in pair-wise comparisons, using label-free quantification by mass spectrometry. Criteria were p<0.05 and fold changes >1.5 or <0.67, as indicated by black lines. Red data-points represent significantly differential phospho-sites, black circles represent those that do not change significantly. The identities of these sites can be found in Supplementary Dataset S2 (D) Chord diagram showing the numbers of identified phospho-sites whose abundance significantly differs upon drug treatment with either IC261 or PF-670462 from their respective controls (>1.5-fold change, p<0.05, n=5), and the overlap between the indicated data sets. Up-regulated peptides are represented in dark red, down-regulated peptides in lighter red.

If CK1 is a core clock component, then we hypothesised that its timekeeping contribution should be phase-dependent. This implies a model whereby normal timekeeping requires oscillating CK1 activity mediated through diurnal activation and nocturnal repression of transcription (rhythmic output) but which also feeds back post-translationally into the extant oscillation (rhythmic input). Strong diurnal transcriptional regulation of CK1 in *O* *. tauri* (Figure 1) could sustain this rhythmicity. To test this hypothesis, cells were subjected to 4-hour inhibition pulses with PF-670462 or IC-261, starting every 2 hours throughout a full circadian cycle. Upon wash-out, phase changes in the resuming rhythm were monitored and plotted on a phase-response curve (Figure 4). A time-of-day-dependent phase delay was observed following pulsed treatment with PF-670462, which was longest towards the end of the day: Inhibition for 4 hours from ZT12 resulted in a ~150 minute delay (Figure 5B, C), and this sensitive phase coincides with the CK1 transcriptional peak (Figure 1). PF-670462 treatment from ZT0 in contrast had no effect on the phase of subsequent rhythms, in line with the lower level of protein expected at that phase based on the transcriptional rhythm. When the same experiment is performed with IC-261, a significant phase-shift would not be expected based on the moderate maximum period lengthening effect this inhibitor elicits (Figure 3A). Indeed, no significant phase-responses are observed with 4-hour pulsed treatments with IC261 (Figure 4A). Together, these results are in line with a model where CK1 activity might indeed contribute rhythmically to normal timekeeping with the functionally critical phase towards the day-to-night transition.

### The CK1-responsive phospho-proteome reveals potentially conserved clock-relevant CK1 targets

Circadian effects of CK1 overexpression or inhibition must result from CK1 activity on as yet unknown clock-relevant targets in this minimal circadian model organism. To identify these CK1 targets, which might be conserved across species, the phospho-proteome of the parent line was compared to that in an overexpression line around the diurnal CK1 peak expression phase (ZT12). Following phospho-enrichment of trypsinised protein extracts of the parent line and CK1-OX8, all peptides for every identified unique phospho-site were quantified using label-free mass spectrometry. Out of all unique phospho-sites (Supplementary Dataset S1), 60 were found to be significantly differential in abundance (fold change > 1.5, p < 0.05, n = 5) between the parent and overexpression line (Figure 5A Supplementary Dataset S1). The observation that the majority of significantly differential sites (52 out of 58) are up-regulated solidifies overexpression of functionally active CK1 protein. Significant overrepresentation of human CK1ε motifs was observed in phospho-sites up-regulated in CK1-OX8 (p=3x10^-5^) compared to non-differential sites (Supplementary Dataset S1), indicating that overexpression indeed results in increased CK1 activity. In addition, this result implies that *O* *. tauri* CK1 acts on motifs similar to clock-relevant human CK1 homolog epsilon.

To identify additional CK1-responsive phosphorylation sites in the *O* *. tauri* minimal proteome, mock-treated cells were compared to IC261- or PF-670462-inhibited cells by label-free proteomic analysis of the phospho-proteome. A further 134 phosphorylation sites were identified as differential between mock-treated and inhibited cells (Figure 5B, 5C and Supplementary Dataset S2). The majority of differential phospho-sites in treated cells is down-regulated compared to the control, in line with inhibition of kinase function by these two compounds. Larger numbers of differentially abundant peptides were identified upon treatment with PF-670462 over IC261, in line with the stronger period lengthening effect observed for this inhibitor.

Plotting all significantly differential phospho-sites on a chord diagram (Figure 5D) reveals that half of the phospho-sites that were down-regulated by *bone fide* CK1 inhibitor IC261 were also repressed by PF-670462 inhibition, implying that targets for both drugs overlap and can be of similar nature. PF-670462 either hits additional clock-relevant kinase functions, or is more effective in inhibiting CK1-responsive events. Either way, the list of phospho-sites differential upon PF-670462 treatment is clock-relevant given the magnitude of the period effect induced by this drug. These combined data provide a unique set of potential clock-relevant targets for CK1, many of which are widely conserved across the tree of life.

## Discussion

Combined results in this manuscript identify CK1 as a clock protein in the plant lineage. A possible reason why CK1 clock function in plants was not previously identified [37,38], might be the high level of functional redundancy found in plants, even in the model plant *Arabidopsis thaliana*. Although different isoforms of CK1 in animals all have clock function, the observation that *Arabidopsis* contains at least 12 CK1 homologs might mean that identifying a clock function by simple genetics is unlikely. The position of *O* *. tauri* CK1 in a phylogenetic tree (Figure 1) indicates that this homolog is closer to the animal and fungal isoforms with known clock function than other plant CK1 proteins are, in line with diversification along the green lineage. The next-closest sequence in the *O* *. tauri* genome, compared to animal CK1s, forms a distant outgroup in the CK1 phylogeny, verifying that indeed only one CK1 isoform exists in this organism.

The observation of period lengthening by both CK1 overexpression (Figure 2) as well as inhibition (Figure 3) appears to be contradictory, as the naive hypothesis would be that these treatments would induce opposite period effects. However, we find that both constitutively heightened CK1 activity by overexpression, as well as constitutively lowered activity by chemical inhibition induces long periodicity, indicating that CK1 activity exhibits complicated effects on timekeeping. In literature, numerous examples exist where the same phenotype was obtained upon overexpression and knock-down of a gene (for an example, see 39 and references therein), although to our best knowledge, this is the first example for a clock-relevant kinase. Phase-dependency of CK1 might go some way in explaining the period-lengthening effect observed upon both treatments, as either inhibition or overexpression represents a constitutive change in activity. A phase response curve of rat behavioural rhythms to PF-670462 was published previously, showing that CK1 activity contributes phase-dependently to sustain rhythms [40]. Results presented here suggest that the requirement for CK1 activity is equally phase-dependent in *Ostreococcus* *tauri* , in near-identical phase compared to rat, with a peak around the day to night transition (Figure 4). Remarkably, a very similar amplitude of ~2.5 hours maximum phase change is observed in both organisms, further supporting the relevance of PF-670462 in *O* *. tauri* , and emphasising conserved action of CK1 in timekeeping across distant clades and different clock outputs.

Additionally, it is now apparent that the kinome constitutes a complex network of intertwined pathways rather than a collection of numerous linear transduction cascades [41]. It is even doubtful whether linear pathways exist in protein biochemistry [42]. Modulation of a hugely important hub kinase like CK1 will have many, practically unforeseeable, results. Complex network effects are mirrored by our observation of down-regulated phosphorylation events upon overexpression of CK1, and by up-regulated sites upon kinase inhibition (Figure 5). These results imply that hypotheses based on a presumption of linear kinase signalling cascades are not fit to explain experimental results described here.

When the phospho-proteome was analysed at the peak of clock activity for CK1 (ZT12), we note that among all CK1-responsive phosphorylation events (Supplementary Dataset S1, S2), an observed phospho-site on *O* *. tauri* Cryptochrome (CRY) is differentially regulated by CK1 inhibition as well as overexpression. Given the established timekeeping roles of CRYs across eukaryotes [2], including *Ostreococcus* [43], its identification here shows that CK1 target proteins identified by the methods described here are likely to include conserved, clock-relevant targets for future study in any organism.

Interestingly a large group of other kinases is differentially phosphorylated upon modified CK1 activity. This result fits well with the notion of a complex kinase network that will not respond linearly to modulation of one of its hub proteins. Five phospho-sites from putative calcium-dependent protein kinases (CDPKs) are differentially regulated by CK1 modulation: 2 are up-regulated upon CK1 overexpression, and 3 are down-regulated in response to PF-670462 treatment. These five sites are from 4 different CDPKs, meaning that one of these proteins (encoded by Ot01g01760) contains one site that is upregulated by CK1 overexpression plus one that is downregulated by CK1 inhibition. In addition, we identified a unique phospho-site derived from a putative mitogen-activated protein kinase (MAPK, Ot08g00430) that is upregulated by overexpression, and downregulated by PF-670462. A second site on the same protein was also downregulated after drug treatment. Several other kinases were found to be differetially phosphorylated, including diacylglycerol kinase and phosphatidylinositol-4 kinase. Other groups of proteins with several differential phospho-sites are involved in transport (such as ion channels, transporters, symporters), transcription (such as helicases, transcription factors, polymerases), or metabolism.

The unicellular marine alga *Ostreococcus* *tauri* is separated from metazoan species by an estimated 1.5 billion years of evolution, which implicates that CK1 is one of the most ancient proteins with a functionally conserved role in timekeeping. Known CK1 targets in other clock systems, like FRQ, PER, CLOCK, and WC2, are not conserved in plants, and although mammalian CK1ε was shown to phosphorylate CRY in culture, this process was dependent on prior phosphorylation of PER [44].

If CK1 is 1) rhythmically regulated by TTFL rhythmicity as a transcriptional clock output, 2) makes a phase-dependent input to the TTFL, and 3) is required for normal timekeeping, it thus becomes indistinguishable from a “core” timekeeping component. We note the parallels with the highly conserved cyclin-dependent kinases (CDKs) of the cell cycle, where CDK activity is under control of the cell cycle through phase-dependent cyclin expression, but in turn regulates cell-cycle progression [45,46], even though transcription factor targets are poorly conserved across the eukaryotes. Just as the CDKs have been hypothesised to be the most ancient and conserved components of the eukaryotic cell cycle, we propose that CK1 constitutes one of the most ancient and conserved mechanistic components that evolved to orchestrate the ancestral eukaryotic circadian networks.

The reduced genomic complexity in *Ostreococcus* *tauri* proved to be instrumental to address basic cell biological questions that are difficult to study in conventional plant model organisms. In line with this argument, addressing the role and targets of CK1 in the biological clock might advance research well beyond the plant field, as CK1 has been shown to be important for many clock-based human diseases and disorders [47-51]. The lists of CK1-responsive phospho-sites reported here provide a useful starting point for subsequent studies into the mechanistic bases of conserved clock-relevant CK1 targets across species, offering tantalising avenues for future investigation into the mechanisms and evolution of cellular circadian rhythms.

## Materials and Methods

All chemicals were purchased from Sigma-Aldrich unless otherwise stated. *Ostreococcus* *tauri* cell culturing and luminescent imaging was performed as described previously [8,33]. In brief, cells were grown in supplemented artificial seawater, under 12/12 light/dark cycles of blue light. Cells were grown for 7 days from a 1/100 split into fresh medium before the start of any experimental conditions. All circadian phase and period analyses were calculated using the mfourfit algorithm on BRASS3 [52].

### Construction of transgenic materials

CK1 (Ot02g06160, gene model XM_003075171.1) was amplified from genomic DNA (aaaaagcaggctacATGTCGTGTGGGCGCGGGCGCG and agaaagctgggtaTTGGTACGCTCGACCTCCGTCG) and cloned into pDONR207 using GateWay technology (Invitrogen). Although other gene models exist for the 5' region, the longest predicted gene was chosen to ensure proper splicing of the transgene. The pOtOX vector [29] was modified by *Sna*B digestion and insertion of a Gateway RfB cassette (Invitrogen) to generate pOtOX-GW and CK1 was recombined from pDONR207 into pOtOX-GW. Vectors were verified using standard sequencing. Transgenic lines were generated as described previously [29,33].

### Drug treatments

Pharmacological experiments were performed using 8 replicates as described previously [8]. Luminescent results from inhibitor-treated cells and overexpression lines were always compared to mock treated or parent cells in the identical well position on a different plate (8 replicates). Washout experiments were performed as in [8].

### Phospho-proteomics

Protein extract (300 µg) for LC-MSMS was prepared as described [31]. After tryptic digestion, samples were cleaned (SupelClean C18 cartridge), dried by SpeedVac, and stored at -20°C. Phospho-peptide enrichment and LC-MSMS analysis was performed as described previously [31]. Acetonitrile and water for LC-MSMS and sample preparation were HPLC quality (Fisher, UK). Formic acid was Suprapure 98-100% (Merck, Darmstadt, Germany) and trifluoroacetic acid was 99% purity sequencing grade. LC-MS label-free quantification was performed using Progenesis 4.0 (Nonlinear Dynamics, UK) as described in [31]. Multicharged ions (2+,3+,4+) were extracted from LC-MS files and MSMS data was searched using Mascot Version 2.4 (Matrix Science Ltd, UK) against the *Ostreococcus* *tauri* subset of the NCBI protein database (12/01/2011; 8,726 sequences) using a maximum missed-cut value of 2, variable oxidation (M), N-terminal protein acetylation, phosphorylation (STY), and fixed carbamidomethylation (C); precursor mass tolerance was 7 ppm and MSMS tolerance 0.4 amu. The significance threshold (p) was <0.05 (MudPIT scoring). A minimum peptide cut off score of 20 was set, corresponding to <3% global false discovery rate (FDR) using a decoy database search. Data were converted and validated using the Pride converter 2 [53] and are available on the public data repository PRIDE (http://www.ebi.ac.uk/pride/), accession numbers 27556-27585. Neutral losses of phosphoric acid typical of serine and threonine phosphorylated were validated manually in all significantly differential phospho-peptides. Ambiguous sites were confirmed by cross-referencing (by sequence, charge, and quantity of residue modifications) with most probable site predictions from MaxQuant [54] version 1.0.13.8 in singlet mode, Mascot settings as above. Where multiple occurrences of residue phosphorylation events were quantified, abundances were summed, collating all charge states, missed cuts and further modifications.

### Statistical analyses

Graphs and statistical analyses were made using GraphPad Prism unless otherwise stated below. CK1 sequences were aligned using Mafft 6 [55], and 3' and 5' non-conserved regions were removed in Jalview [56]. Maximum Likelihood was employed to infer phylogeny (using RAxML 7.2.8-α [57]). No evolutionary root was assumed *a priori*, and a gamma model of evolutionary rate heterogeneity was used (α = 0.67). Amino acid replacement scoring was determined using the WAG matrix [58]. Support for branches was evaluated using bootstrap analysis with 1000 starting trees.

For statistical analyses on the phospho-proteomics results, data were tested at the phosphorylation site level (see Supplementary Datasets S1 and S2). Abundances were arcsinh transformed to generate normal distributions. Phospho-sites with a significantly differential mean-abundance, compared to the control, were identified with a two-tailed t-test for independent samples. Within group means on raw values were calculated to determine the fold changes. Peptides with p<0.05 and fold change ratio >1.5 between groups were defined as significantly differential.

To predict CK1 target sites, GPS 2.1 [59] was used on the v2.0 assembly of the *Ostreococcus* *tauri* genome [27,60]. Peptides were aligned to the reference assembly and CK1 targets were transferred. Highly conservative cut-off threshold was used for the CK1ε classifier. Permutation-tests were used to test if the mean fold-change of CK1ε targets was significantly different to the remaining peptides in the dataset. The significance of over- or under-representation of CK1 motifs was estimated using a Monte Carlo permutation test. 10 million permutations of the observed partition dimensions were re-sampled and the two-tailed p-value was estimated as the frequency with which the difference in the mean fold-change of the random permutations exceeded the observed difference.

The co-occurrence of unique residue phosphorylation events was enumerated across all peptides between all statistically significant groups. The resulting co-occurrence of phosphorylation events was visualised using the chord visualization [61] from the D3 library (http://d3js.org).

## Supporting Information

Dataset S1 Identified phospho-sites in the overexpression line versus parent line, with identification and quantification details. (XLS)

Dataset S2 Identified phospho-sites in CK1-inhibited cells versus mock-treated cells, with identification and quantification details. (XLS)
